# *Vasa*-Like DEAD-Box RNA Helicases of *Schistosoma mansoni*

**DOI:** 10.1371/journal.pntd.0001686

**Published:** 2012-06-12

**Authors:** Danielle E. Skinner, Gabriel Rinaldi, Sutas Suttiprapa, Victoria H. Mann, Pablo Smircich, Alexis A. Cogswell, David L. Williams, Paul J. Brindley

**Affiliations:** 1 Department of Microbiology, Immunology & Tropical Medicine, and Center for Neglected Diseases of Poverty, School of Medicine & Health Sciences, George Washington University, Washington, D. C., United States of America; 2 Departamento de Genética, Facultad de Medicina, Universidad de la República, (UDELAR), Montevideo, Uruguay; 3 Department of Pathobiology, Faculty of Science, Mahidol University, Bangkok, Thailand; 4 Department of Microbiology and Immunology, Rush University Medical Center, Chicago, Illinois, United States of America; University of Queensland, Australia

## Abstract

Genome sequences are available for the human blood flukes, *Schistosoma japonicum*, *S. mansoni* and *S. haematobium*. Functional genomic approaches could aid in identifying the role and importance of these newly described schistosome genes. Transgenesis is established for functional genomics in model species, which can lead to gain- or loss-of-functions, facilitate vector-based RNA interference, and represents an effective forward genetics tool for insertional mutagenesis screens. Progress toward routine transgenesis in schistosomes might be expedited if germ cells could be reliably localized in cultured schistosomes. Vasa, a member of the ATP-dependent DEAD-box RNA helicase family, is a prototypic marker of primordial germ cells and the germ line in the Metazoa. Using bioinformatics, 33 putative DEAD-box RNA helicases exhibiting conserved motifs that characterize helicases of this family were identified in the *S. mansoni* genome. Moreover, three of the helicases exhibited *vasa*-like sequences; phylogenetic analysis confirmed the three *vasa*-like genes—termed *Smvlg1*, *Smvlg2*, and *Smvlg3*—were members of the Vasa/PL10 DEAD-box subfamily. Transcripts encoding *Smvlg1*, *Smvlg2*, and *Smvlg3* were cloned from cDNAs from mixed sex adult worms, and quantitative real time PCR revealed their presence in developmental stages of *S. mansoni* with elevated expression in sporocysts, adult females, eggs, and miracidia, with strikingly high expression in the undeveloped egg. Whole mount *in situ* hybridization (WISH) analysis revealed that *Smvlg1*, *Smvlg2* and *Smvlg3* were transcribed in the posterior ovary where the oocytes mature. Germ cell specific expression of schistosome *vasa*-like genes should provide an informative landmark for germ line transgenesis of schistosomes, etiologic agents of major neglected tropical diseases.

## Author Summary

Schistosomes, the blood flukes, are responsible for the major neglected tropical diseases termed schistosomiasis, which afflicts >200 million people in impoverished regions of the developing world. The genome sequence of these parasites has been decoded recently. The DEAD-box family is the largest of RNA helicase families. These enzymes play roles in RNA metabolic processes—transcription, pre-mRNA splicing, ribosome biogenesis, transport, initiation of translation, organelle gene expression, and RNA decay. Database searches indicated that *S. mansoni* has at least 33 DEAD-box helicases. A DEAD-box helicase known as Vasa is a determinant in germ line segregation and maintenance. Three schistosome DEAD-box RNA helicases exhibited Vasa-like sequences, and phylogenetic analysis confirmed they were members of the Vasa/PL10 DEAD-box subfamily. Quantitative real time PCR revealed that all three, termed *Smvlg1*, *Smvlg2*, and *Smvlg3*, were expressed in developmental stages of *S. mansoni*, with elevated expression in adult females, eggs, miracidia and sporocysts. Moreover, whole mount *in situ* hybridization analysis confirmed that these *vasa*-like genes were expressed in the schistosome ovary. These findings provide insights into the mechanisms underlying development of germ cell lines and gonads in schistosomes and have implications for understanding the role of RNA helicases, germ and stem cell biology.

## Introduction

Schistosomiasis is considered the most important of the human helminthiases in terms of morbidity and mortality. It is endemic to 76 countries, affecting an estimated 200 million people with an additional 700 million people at risk of infection [1]–[3]. Draft genome sequences of all three of the major schistosome species are now available [4]–[7], underscoring a pressing need to develop functional genomic approaches to identify the role and importance of schistosome genes that might be targeted for development of novel anti-schistosomal interventions.

Retrovirus-mediated transgenesis is an established functional genomics approach for model species, e.g. [8]. It offers the means to establish gain- or loss-of-function phenotypes, can facilitate vector-based RNA interference, and represents a powerful forward genetics tool for insertional mutagenesis screens. Although murine leukemia virus (MLV) pseudotyped with vesicular stomatitis virus glycoprotein (VSVG) mediates somatic transgenesis in *S. mansoni* [9], vertical transmission of integrated transgenes has not been reported in schistosomes. A potential route to the germ line in schistosomes is to transduce the egg, and genetic manipulation of this developmental stage with MLV is feasible [10]. In addition, germ line transgenesis might be more readily monitored if schistosome germ cells could be localized and tracked in cultured forms of this pathogen.

Vasa, an adenosine 5′-triphosphate (ATP)-dependent DEAD-box RNA helicase, is an archetypal, metazoan germ cell specific marker [11], [12]. The highly conserved *vasa* gene has been studied widely as a germ cell marker in *Caenorhabditis elegans*, *Drosophila melanogaster*, *Xenopus* species, *Danio rerio*, *Mus musculus* and other species [13]–[22]. Reporter gene-*vasa* transgenic lines have been developed to isolate germ line cells [23]–[25]. Moreover, *vasa*-like homologues have been reported in Platyhelminthes including *Macrostomum lignano*, *Schmidtea polychroa*, several *Dugesia* species, *Neobenedenia girellae*, and *Paragonimus westermani* [11], [26]–[31].

Here, *vasa*-like genes in *S. mansoni* were identified and their developmental expression examined. Whole mount *in situ* hybridization and real-time quantitative PCR revealed specific expression in the schistosome ovary and strikingly elevated expression in the developing egg. Since Vasa is a reliable germ cell marker in other flatworms, we anticipate that reporter transgenes vectored by an integration competent vector such as MLV and driven by a *vasa* promoter might be of use to monitor transgene integration into schistosome germ cells.

## Materials and Methods

### Ethics statement

Female Swiss-Webster mice infected with *S. mansoni* were obtained from the Biomedical Research Institute (BRI), Rockville, MD and housed at the Animal Research Facility of the George Washington University Medical Center, which is accredited by the American Association for Accreditation of Laboratory Animal Care (AAALAC no 000347) and has an Animal Welfare Assurance on file with the National Institutes of Health, Office of Laboratory Animal Welfare, OLAW assurance no. A3205-01. All procedures employed were consistent with the Guide for the Care and Use of Laboratory Animals. Maintenance of the mice and subsequent recovery of schistosomes were approved by the Institutional Animal Care and Use Committee (protocol approval no. A137) of the George Washington University.

### Schistosomes

Mice and *Biomphalaria glabrata* snails infected with the NMRI (Puerto Rican) strain of *S. mansoni* supplied by Dr. Fred Lewis, Biomedical Research Institute, Rockville, MD.

Eggs were recovered from livers of infected mice [32]. Aliquots of eggs were harvested while the remaining eggs were washed three times with 1×PBS supplemented with 2% penicillin, streptomycin, fungizone and transferred to sterile water under a bright light to induce egg hatching [33]. Newly hatched miracidia were snap frozen or transferred to sporocyst media and cultured as primary sporocysts for up to 15 days, as described [33]. *In vitro* laid eggs (IVLE) were obtained as described [34]. In brief, adult *S. mansoni* recovered from mice by portal perfusion were transferred to six-well culture plates with mesh inserts of 74 µm pore diameter, containing Basch's medium at 37°C under 5% CO_2_ (see [33]). Media were replaced twice a day. IVLE fell through the pores whereas the adult worms remained on the mesh. At 48 hours, the IVLE were harvested by filtering media containing the eggs through a six-well culture plate insert of 0.8 µm pore size (Greiner Bio-One, Art. No. 657638). After concentrating the IVLE, aliquots of IVLE were harvested at several intervals - 12 hours, 2, 4, 6, and 7 days, during which time IVLEs matured, as described [35]. After perfusion from mice, other male and female adult *S. mansoni* worms were separated, washed three times with 1×PBS, pH 7.4, and stored at −80°C. Cercariae released from infected *B. glabrata* snails were transformed mechanically into schistosomula [33], and cultured for 10 days in Basch's medium [36] at 37°C under 5% CO_2_ in air.

### Database searches and sequence analysis

To search for *S. mansoni vasa*-like genes, Pfam searches of the draft genome of S. *mansoni* at Wellcome Trust Sanger Institute GeneDB, http://www.genedb.org/Query/pfam?taxonNodeName=Smansoni were carried out using DEAD/DEAH helicase (Pfam accession PF00270) and eukaryotic RNA helicase (Pfam accession PF00271) domains that included DEAD/DEAH helicases along with other RNA helicases as queries [37]. The Pfam database uses the profile hidden Markov model software, HMMER3. Separately, the genome was screened using the keyword ‘DEAD’ as a query in a motif search at GeneDB, http://www.genedb.org/Query/motif?taxonNodeName=Smansoni. Putative orthologous proteins for each *S. mansoni* DEAD-box gene were predicted by protein BLAST at NCBI with the current default settings, including, analysis against all databases using Blosum 62 scoring matrix with the following criteria: at least 20% identity to the query sequence and *e*-value lower than 0.001. Pairwise comparisons were performed using the pairwise BLAST program at NCBI, http://blast.ncbi.nlm.nih.gov/ to determine identity/similarity percentages.

### Bioinformatics characterization of schistosome *vasa*-like genes

The deduced amino acid sequences of three putative *S. mansoni* DEAD-box helicases were aligned with reference sequences from informative species by ClustalW (Bioedit) software [38]. Phylogenetic analysis was conducted in MEGA5 [39]; 30 amino acid sequences were included in the analysis. The sequences were aligned in Bioedit and gaps were excluded from the alignment. A bootstrapped Neighbor-joining tree was generated based on the whole deduced amino acid sequences, with the eukaryotic initiation factor-4A (eIF4A) as the out-group [40]. Bootstrap analysis was performed with 1,000 data sets. Bootstrap values shown on the tree represent the percentage of replicate trees in which the associated taxa clustered together. The tree was drawn to scale, with branch lengths in the same units as those of the evolutionary distances used to infer the phylogenetic tree. The evolutionary distances were computed using the p-distance method, with distances in units of the number of amino acid substitutions per site. Sub-cellular sites of locations of proteins were predicted using PSORT II, http://psort.ims.u-tokyo.ac.jp/ [41]. In addition, gene structures including exon-intron boundaries were examined in order to predict evolutionary relationships among the schistosome vasa orthologues. Using GeneDB accessions Smp_033710, Smp_154320 and Smp_068440,and cDNA sequences JQ619869– JQ619871 (below), along with exon structures displayed by Artemis, we predicted exon-intron boundaries including positions of motifs characteristic of Vasa encoded by exons. Exon-intron boundaries were displayed using FancyGene [42].

### Gene expression analysis

Total RNA was isolated from *S. mansoni* developmental stages using the RNAqueous®-4PCR kit (Ambion, Austin TX). Residual DNA contaminating the RNA was removed by digestion with RNase-free DNase I (TurboDNase, Ambion) at 37°C for 1 h. cDNAs were synthesized using the iScript™ cDNA synthesis kit (Bio-Rad, Hercules CA) using 75 ng of total RNA as the substrate. Relative expression of genes of interest was analyzed by quantitative RT-PCR (qRT-PCR). TaqMan probes and qPCR primers were designed with the assistance of Beacon Designer (Premier Biosoft International, Palo Alto, CA): *Smvlg1* forward primer, 5′-ACG ACT ATA ATG AGA ATA ATC TTG-3′; *Smvlg1* reverse primer, 5′-CCA AAC TTT ATG TGC CTC-3′; *Smvlg1* probe, 5′-/56-FAM/GTT CAG ATG GTG GTG GT/3IABlk_FQ/-3′; *Smvlg2* forward primer, 5′-TGA GGC TAT AAC ACT TAT TC-3′; *Smvlg2* reverse primer, 5′-CCT TCT TGA ATT TCC ATA TTG-3′; *Smvlg2* probe, 5′-/56-FAM/AAG CAA CAA CCA TCA AGC AGA ACA/3IABlk_FQ/-3′; *Smvlg3* forward primer, 5′-TAC CGT TCC AAC ATT AAG-3′; *Smvlg3* reverse primer, 5′-GTT CCA GAC TCA CTT TAC-3′; *Smvlg3* probe 5′-56-FAM/CTG CTC ACC ATA TCA ACA AGA CGC/3IABlk_FQ/-3′. TaqMan probe and primers targeting the schistosome *alpha tubulin* (GenBank XM_002581074; SCMSAT1A), as a reference gene were: *SmAtubulin* forward primer, 5′-GTG CTG TAT GTA TGT TAA GTA-3′; *SmAtubulin* reverse primer, 5′-CGT GCT TCA GTA AAT TCA-3′; and *SmAtubulin* probe, 5′-56-FAM/TCT TCC ATA CCT TCA CCA ACA TAC CAA/3IABlk_FQ/-3′. Quantitative PCRs were performed, with duplicate biological replicates each with triplicate technical replicates. Reactions were carried out in 20 µl volumes with primer-probe sets and Perfecta qPCR FastMix, UNG (Quanta Bioscience, Gaithersburg, MD). The qPCR conditions included an initial denaturation at 95°C for 3 min followed by 40 cycles of 30 s at 95°C and 30 s at 55°C, performed in a thermal cycler (iCycler, Bio-Rad) and a Bio-Rad iQ5 detector to scan the plate in real time. Relative quantification was calculated following the 2^−ΔΔCt^ method [43]. *Alpha tubulin* was employed as the reference because it maintains steady expression levels among developmental stages [44] and the cercarial stage was used as a calibrator.

### Whole mount *in situ* hybridization

Whole mount *in situ* hybridization (WISH) to adult *S. mansoni* worms was carried out using the method of Cogswell and colleagues [45], adapted from methods for planarians [46]. Each experimental group included ∼20 female and ∼20 male adult schistosomes; the experiment was repeated three times. In brief, schistosomes were fixed in 4% paraformaldehyde (PFA), adult males were reduced with 50 mM dithiothreitol, 1% NP-40, and 0.1% SDS, and both males and females were dehydrated in a methanol series, then stored at −20°C. Prior to WISH, worms were bleached with 6% H_2_O_2_, rehydrated through a methanol series to 1×PBS with 0.3% Triton-X 100, permeabilized using proteinase K, and re-fixed in 4% PFA. Hybridization was performed at 56°C with digoxigenin [DIG]-labeled riboprobes (below) for 18–20 h. Incubation in anti-DIG antibody at 1∶2000 diluted in blocking solution was performed overnight at 4°C, and signals developed with nitro blue tetrazolium/5-bromo-4-chloro-3-indolylphosphate. WISH specimens, mounted in glycerol, were examined using a Zeiss Axio Observer A.1 inverted microscope fitted with a camera AxioCam ICc3 camera (Zeiss). Manipulation of digital images was undertaken with assistance of AxioVision release 4.6.3 (Zeiss) and ImageJ 1.45 software [47]. Manipulations were limited to insertion of scale bars, adjustments of brightness and contrast, cropping and the like. Image enhancement algorithms were applied in linear fashion across the entire image and not to selected aspects.

Transcripts encoding *Smvlg1*, *Smvlg2*, and *Smvlg3* (below) were amplified from mixed-sex, adult worm cDNA with the following primers: *Smvlg1* cDNA forward primer, 5′- ATG TCT TAC GAC TAT AAT GAG AAT A-3′ and *Smvlg1* cDNA reverse primer, 5′-CTA ATT GCC CCA CCA GTC TGG AGA A-3′, PCR product size 1,914 bp; *Smvlg2* cDNA forward primer, 5′-ATG AAT AGA GTA ATT CTG AGT CAG C-3′ and *Smvlg2* cDNA reverse primer, 5′-TCA CAA ATA TCG CAT CAA ACT ATC A-3′, PCR product size 1,839; *Smvlg3* cDNA forward primer, 5′-ATG GAG AGC CTA GAA AAC AAT TTT GGC-3′ and *Smvlg3* cDNA reverse primer, 5′- AAA CAT CAA AGT CTG TCT TTT TC-3′, PCR product size 1,065 bp of the 2,835 bp complete cDNA. PCR products were cloned into vector pCR4-TOPO (Invitrogen) and the nucleotide sequences of the inserts determined. These sequences have been assigned GenBank accessions JQ619869–JQ619871 for *Smvlg1*, *Smvlg2*, and *Smvlg3*, respectively. Previously, we have described Argonaut 2 (Ago2) cloned into pJC53.2 [45].

Antisense and sense digoxigenin-labeled riboprobes were synthesized by *in vitro* transcription employing PCR products as templates using gene specific primers tailed with the T7 promoter sequence as follows, (T7 promoter sequence italicized): *Smvlg1* sense forward primer, 5′- *TAA TAC GAC TCA CTA TAG GG*C AAA CGG TTC AGA TGG TGG TGG TGC C-3′ and *Smvlg1* sense reverse primer, 5′- GCG CTT ATA GAA TCA CCA GGA CCT TGC -3′, spanning coding DNA positions 62–737; *Smvlg1* antisense forward primer, 5′-CAA ACG GTT CAG ATG GTG GTG GTG CC-3′ and *Smvlg1* antisense reverse primer, 5′- *TAA TAC GAC TCA CTA TAG GG*G CGC TTA TAG AAT CAC CAG GAC CTT GC -3′, spanning coding DNA positions 62–737; *Smvlg2* sense forward primer, 5′-*TAA TAC GAC TCA CTA TAG GG*T TAC TTT ACA TTC ATC ACG TCA TA-3′ and *Smvlg2* sense reverse primer, 5′- CTA TAA AAT GAC ATC CCT TAT TCA AC -3′, spanning coding DNA positions 32–769; *Smvlg2* antisense forward primer, 5′-TTA CTT TAC ATT CAT CAC GTC ATA-3′ and *Smvlg2* antisense reverse primer, 5′- *TAA TAC GAC TCA CTA TAG GG*C TAT AAA ATG ACA TCC CTT ATT CAA C -3′, spanning coding DNA positions 32–769; *Smvlg3* sense forward primer, 5′- *TAA TAC GAC TCA CTA TAG GG*T ATC TGA GCT TAA GAG ATG CGT CC-3′ and *Smvlg3* sense reverse primer, 5′-ACA TGC CAT CAA ATC ACG TTT-3′, spanning coding DNA positions 28–606; *Smvlg3* antisense forward primer, 5′-TAT CTG AGC TTA AGA GAT GCG TCC-3′, and *Smvlg3* antisense reverse primer, 5′-*TAA TAC GAC TCA CTA TAG GG*A CAT GCC ATC AAA TCA CGT TT-3′, spanning coding DNA positions 28–606; *Ago2* antisense forward primer, 5′-CCA GTG AAA GTC GTT GCA GA-3′ and *Ago2* antisense reverse primer, 5′-*TAA TAC GAC TCA CTA TAG GG*A CTT GCG GAC TTG CTG AGT T-3′, spanning coding DNA positions 955–2,149. The cycling protocol included an initial denaturation at 95°C for 2 min followed by 35 cycles of 30 s at 95°C, 30 s at 55°C, 60 s at 72°C, and a final extension at 72°C for 10 min. Riboprobes labeled with digoxigenin-11-UTP (Roche) were synthesized using the T7 polymerase Riboprobe System (Promega).

## Results

### 33 putative DEAD-box RNA helicases in the *Schistosoma mansoni* genome

The presence of genes encoding the conserved DEAD/DEAH helicase (PF00270) or eukaryotic RNA helicase (PF00271) domains in the genome of *S. mansoni* was investigated by sequence query and by keyword searches. Positive hits were analyzed for presence of the DEAD-box motif, Asp-Glu-Ala-Asp, with degeneracy permitted in the third residue of the motif, and for nine other motifs diagnostic of DEAD-box RNA helicases [48], [49]. However, positive hits bearing a DExH-box motif (Asp-Glu-x-His) were excluded from subsequent analyses [48], [50]. This search identified 33 putative RNA helicases that by BLAST exhibited *e*-values≤2e^−69^ and percent identities of 39–99%, thus suggesting they were DEAD-box proteins (Table S1). A multiple sequence alignment of these 33 orthologues revealed that all ten diagnostic motifs of DEAD-box RNA helicases were mostly conserved although, for example, motifs V and V1 were absent from Smp_034190.2, Smp_095920, Smp_096530, and Smp_166400, (Figure S1; Table S1).

### *Vasa*-like genes of *S. mansoni*

Among the 33 DEAD-box helicases of *S. mansoni*, BLAST revealed that three of the deduced schistosome enzymes, *Smvlg1*, *Smvlg2* and *Smvlg3* (GeneDB accessions Smp_033710, Smp_154320 and Smp_068440) (GenBank JQ619869, JQ619870, JQ619871) showed high identity to Vasa proteins from other flukes: *Smvlg1* was 89% identical and 95% similar to the DDX3X ATP-dependent RNA helicase of the blood fluke *S. japonicum* (GenBank CAX73517), and was 74% identical and 84% similar to the ATP-dependent RNA helicase of the human liver fluke *Clonorchis sinensis* (GenBank GAA28330); *Smvlg2* was 51% identical and 68% similar to the DDX3/DED1 ATP-dependent RNA helicase of *C. sinensis* (GenBank GAA56795), and 44% identical and 63% similar to the *N. girellae vasa-like 2* (*Ngvlg2*) protein (GenBank BAF44660); *Smvlg3* was 90% identical and 94% similar to the Vasa of *S. japonicum* (GenBank AFC17964), and 60% identical and 72% similar to the partial ATP-dependent RNA helicase sequence, DDX3X, of *C. sinensis* (GAA39366). *Smvlg1* spanned 9,569 bp (CABG01000013; chromosome 2 unplaced supercontig 0108) where the gene included nine exons that encoded 637 deduced amino acid residues. *Smvlg2* spanned 12,752 bp (CABG01000090; supercontig 0203); the gene included eight exons that encoded 625 deduced amino acid residues. *Smvlg3* spanned 15,318 bp (HE601625; chromosome 2; supercontig Smp_scaff000223); the nine exons encoded 944 deduced amino acid residues.

**Figure 1:**
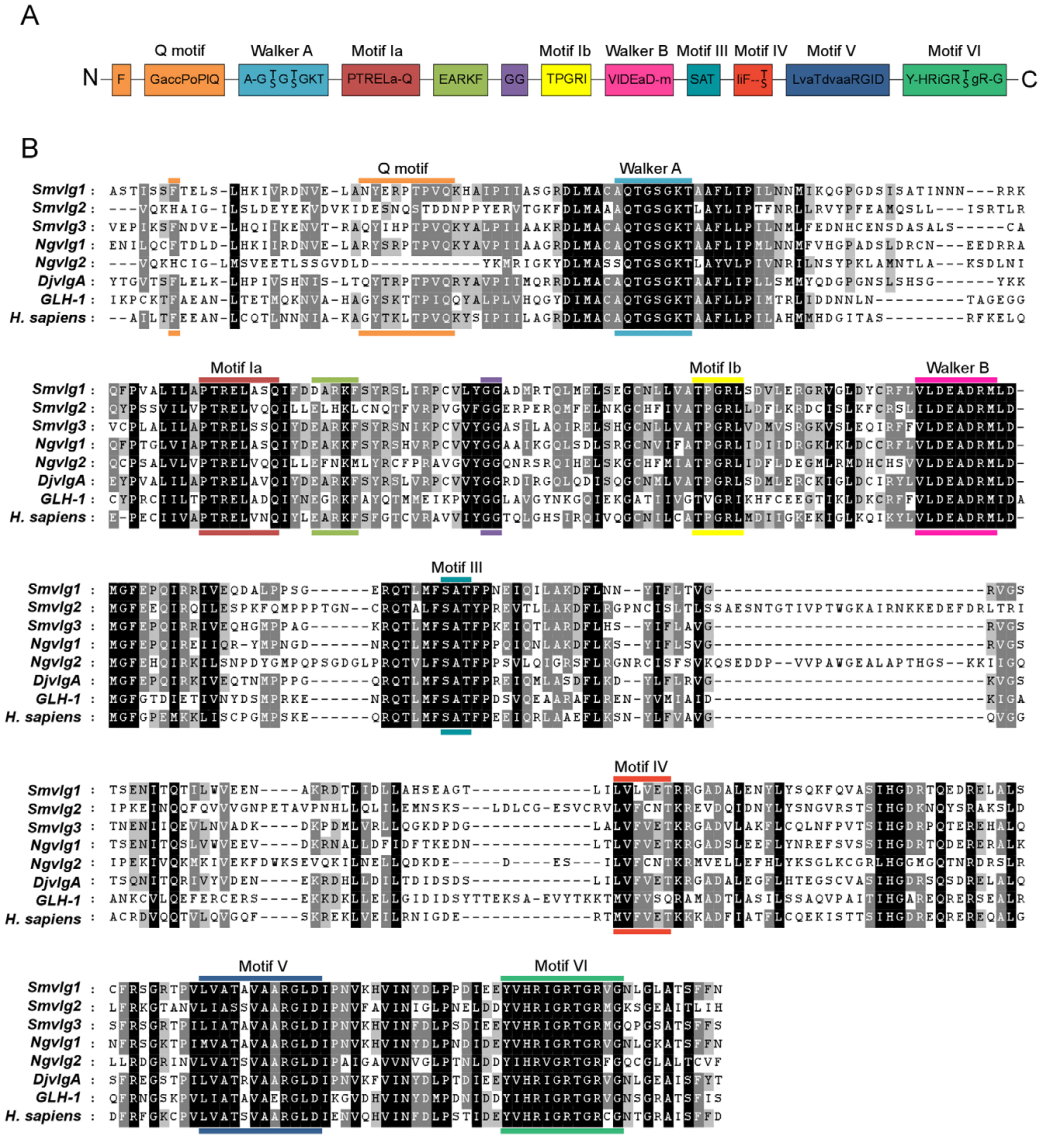
Multiple sequence alignment of DEAD-box RNA helicases from *Schistosoma mansoni*. Panel A: Schematic representation of ten motifs conserved in DEAD-box helicases; the EARKF motif, also shown, is conserved in Vasa and PL10 DEAD-box helicases. For each motif, with the exception of the Q motif, the capital case letters in the consensus sequence indicate highly conserved amino acids (in >80% enzymes) whereas lower case letters represent amino acids conserved in 50–79% of helicases [49]. The capital case letters of the Q motif indicate amino acids conserved between 49%–99% of helicases. The lower case letters represent groups of amino acids, where a represents an aromatic residue, c is a charged residue, o is an alcohol, and l is an aliphatic residue [48]. Panel B: Multiple sequence alignment of DEAD-box RNA helicases from *Schistosoma mansoni*, informative Vasa-like enzymes from other flatworms, *Caenorhabditis elegans* and *Homo sapiens*. DEAD box motifs are indicated with colored lines above and below the sequence that corresponds with the colors in the schematic representation (panel A). Accession numbers of aligned orthologues: *Smvlg1* of *Schistosoma mansoni* (JQ619869), *Smvlg2* of *S. mansoni* (JQ619870), *Smvlg3* of *S. mansoni* (a consensus of Smp_068440 and JQ619871), *Ngvlg1* of *Neobenedenia girellae* (BAF44659), *Ngvlg2* of *N. girellae* (BAF44660), *DjvlgA* of *Dugesia japonica* (BAA34993), GLH-1 of *Caenorhabditis elegans* (P34689.3), and Vasa of *Homo sapiens* (AAF72705).

Multiple sequence alignment of the three *S. mansoni* Vasa-like proteins revealed that, with the exception of *Smvlg2*, they exhibited the ten motifs characteristic of DEAD-box proteins [51], [52] (Figure 1). The Q motif (GaccPoPIQ; see key in Figure 1) and the conserved Phe upstream of Gln, were absent from the *Smvlg2* protein, in like fashion to *Ngvlg2* of the monogenean *N. girellae* [28], [53]. In addition to the ten conserved DEAD-box motifs, an EARKF motif found only in Vasa, PL10, and An3 [30], [54], [55] was present in *Smvlg1* as DARKF (residues 275–279) and in *Smvlg3* as EARKF (residues 259–263). This motif was absent from *Smvlg2* (Figure 1B).

Beyond conserved motifs of the core region, the alignment revealed similarities in the amino and carboxy termini of the schistosome Vasa proteins. The amino-terminus of *Smvlg1* is glycine-rich (13% Gly residues 23–111), including five Arg-Gly repeats and one RGG motif. In *Smvlg3*, asparagine-rich stretches occurred at the COOH-terminus. Moreover, in both *Smvlg1* and *Smvlg3* the characteristic Trp and Asp residues occurred proximal to the stop codon [56]. PSORT II analysis predicted that *Smvlg1* and *Smvlg3* were nuclear proteins (PSORT II scores, 60.9–69.6%) whereas *Smvlg2* Vasa-like protein appeared to be located in the mitochondrion (score, 65.2%).

**Figure 2:**
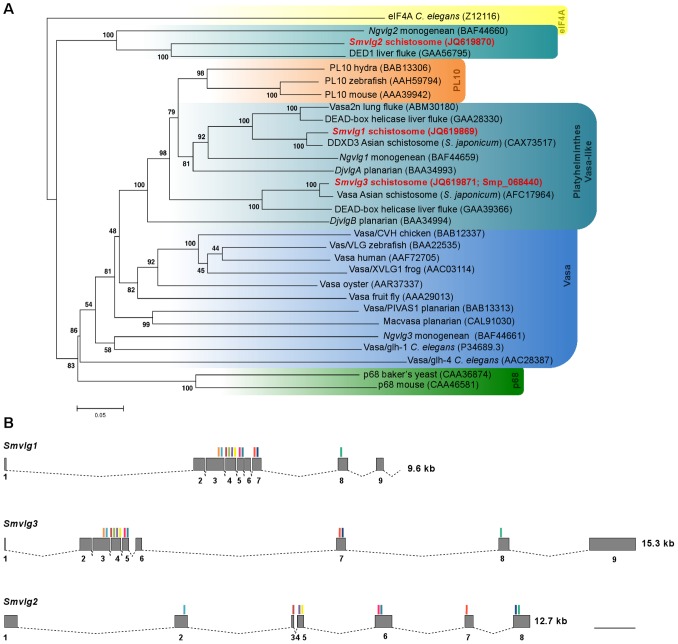
Phylogenetic tree and gene structure relationships of *Schistosoma mansoni* DEAD-box RNA helicases. Panel A: Evolutionary relationships of Vasa, PL10, p68, and eIF4A proteins among informative species were inferred using the Neighbor-Joining method based on entire protein sequences. The *S. mansoni* vasa like genes are shown in red ink. Among DEAD-box helicases p68 is closely related to Vasa and PL10. IF4A served as the outgroup. Branch names indicate the common name of the species displayed; GenBank and GeneDB accessions are provided. Colored boxes are used to highlight families of DEAD box helicases, e.g. the blue region includes vasa orthologues, and the teal boxes include platyhelminth Vasa-like enzymes, etc. Panel B: Exon-intron boundary map revealed evolutionary relationships among *Smvlg1*, *Smvlg2* and *Smvlg3*. Grey blocks represent exons and the black dashed-lines represent introns. Numbers below blocks denote the exon number. Vertical bars above the exons indicate sites encoding the DEAD-box characteristic motifs. Colors of the vertical bars correspond with the colors in the schematic representation of the DEAD-box motifs presented in Figure 1A. Scale bar, 1 kb.

Phylogenetic analysis confirmed that *Smvlg1* belonged to the PL10 family. However, it grouped within a separate cluster with orthologues from other flatworms classified as *vasa*-like genes (Figure 2). *Smvlg2* grouped with *Ngvlg2* of the monogenean *N. girellae* which expression and RNAi analyses had confirmed to be a *vasa*-like gene, although phylogenetic analysis had indicated that it was closely related to the Vasa subfamily or p68 family [28], and the DDX3/DED1 ATP-dependent RNA helicase of *C. sinensis*. *Smvlg3*, like *DjvlgB* of the planarian *D. japonica*, appeared to be a member of the PL10 subfamily. *Smvlg3* most closely clustered with the Vasa of *S. japonicum* and DDX3X of *C. sinensis*.

Also, we examined gene structure and exon-intron boundaries for the three schistosome *vasa*-like genes. This revealed apparently closer evolutionary relationship between *Smvlg1* and *Smvlg3*, and more distant relation to *Smvlg2* (Figure 2B), which in turn supports the relationships established in the phylogenetic analysis (Fig. 2A). *Smvlg1* and *Smvlg3* shared identical numbers of exons and clear synteny of gene fragments encoding the characteristic DEAD-box motifs (Fig. 2B).

### Elevated expression of *vasa*-like genes in the developing egg

**Figure 3:**
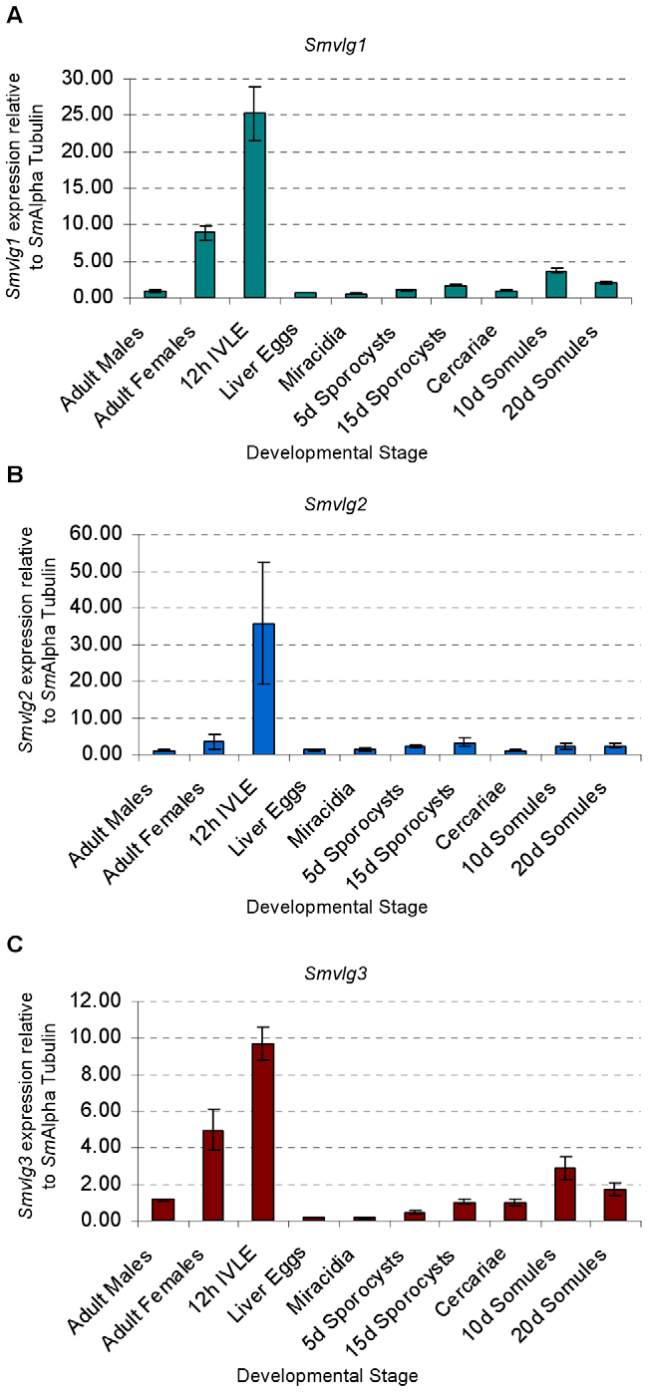
Developmental expression of Vasa-like DEAD-box helicases in *Schistosoma mansoni*. Expression levels of *Smvlg1 (Panel A), Smvlg2 (B)* and *Smvlg3 (C)* of developmental stages of *S. mansoni* were determined by quantitative real time PCR. Normalized fold expression of the *vasa*-like genes is presented; bars represent one standard deviation of the mean.

Developmental expression of the *vasa*-like genes was investigated using qRT-PCR. Transcripts encoding *Smvlg1*, *Smvlg2*, and *Smvlg3* were detected in all of developmental stage of *S. mansoni* examined (Figure 3). However, much higher levels of expression were detected in 12 hour IVLE (often >10 times higher than in other stages) and to a lesser extent in adult females. Generally, patterns of expression of each of the three genes were similar among the developmental stages (Figure 3, panels A, B and C).

### Schistosome *vasa* genes exhibit ovary specific expression

**Figure 4:**
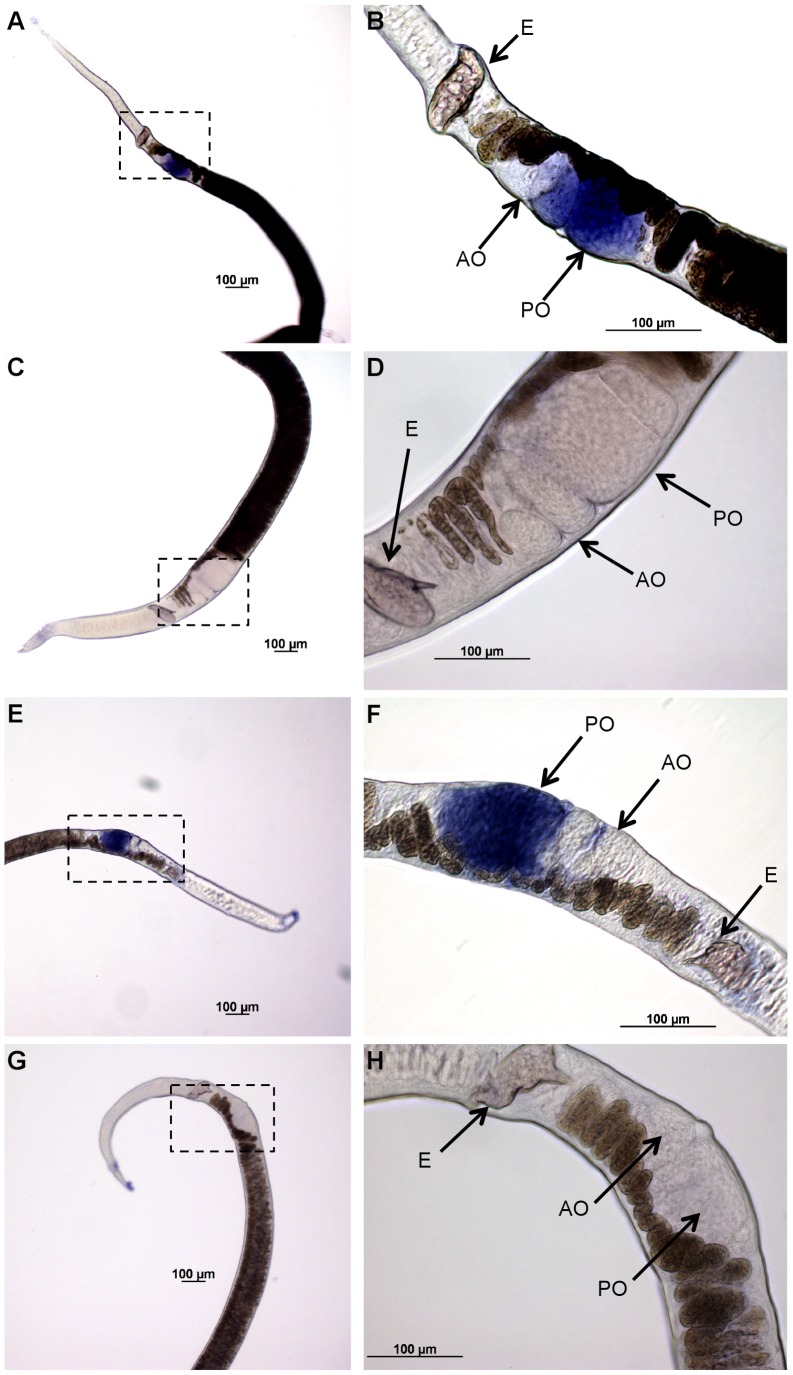
Localization of *Smvlg1* and *Smvlg2*in adult *Schistosoma mansoni* females. Riboprobes specific for *Smvlg1 and Smvlg2* localized to the posterior ovary of *S. mansoni*. A and B: Female worms hybridized with *Smvlg1* antisense riboprobe showing staining in the posterior region of the ovary. B, higher magnification view of the boxed area in image A. C and D: Female worms hybridized with *Smvlg1* sense riboprobe revealing absence of staining; D, higher magnification view of the boxed area in panel C. E and F: Female worms hybridized with *Smvlg2* antisense riboprobe showing staining in the posterior region of the ovary. F, higher magnification view of the boxed area in panel E. G and H: Female worms hybridized with *Smvlg2* sense riboprobes. H, higher magnification view of the boxed area in panel G. Abbreviations: AO, anterior ovary; E, egg; PO, posterior ovary. Scale bars, 100 µm.

To determine sites of expression of *Smvlg1*, *Smvlg2*, and *Smvlg3* in adult schistosomes, adult *S. mansoni* worms were examined in whole mount *in situ* hybridization (WISH). WISH probed with digoxigenin-labeled riboprobes revealed that expression of each of *Smvlg1*, *Smvlg2*, and *Smvlg3* was confined to the ovary of the adult females (Figure 4; and *Smvlg3* not shown), this pattern of expression was observed in the antisense treated worms, but not in the sense treated worms. All or most of the ∼20 female worms examined in each of the three replicates of the WISH analysis were positive for antisense probes, for each of the three *vasa*-like genes. In females, transcripts were localized to the posterior region of the ovary where mature oocytes develop. Cognate sense strand probes were hybridized in parallel; signals in the ovary were not detected using any of the sense probes (Figure 4; and *Smvlg3* not shown). In addition, anti-sense probe signals were not evident after hybridization to the testes or other sites in male worms (not shown). An antisense probe to *Argonaut2* (*Ago2*) was used as a positive control for WISH (not shown) (see [45]). The three *Smvlg* probes targeted the 5′-region of the transcripts, which do not encode diagnostic DEAD-box motifs.

## Discussion

The DEAD-box family is the largest of RNA helicase families, belonging to the helicase superfamily II. These enzymes are involved in RNA metabolic processes - transcription, pre-mRNA splicing, ribosome biogenesis, RNA transport, translation initiation, organelle gene expression, and RNA decay [48], [57]. DEAD-box helicases are over-expressed in cancer cells, further indicating roles in diverse cellular processes [58], [59]. Vasa, an adenosine 5′-triphosphate (ATP)-dependent DEAD-box RNA helicase, is an archetypal, perhaps indispensable, metazoan germ cell specific marker [11], [12]. As befits a founding member of the DEAD-box RNA helicase family, Vasa exhibits nucleic acid unwinding activity especially as a translational regulator in determination and maintenance of germ cells [12], [60]. For example, in *D. melanogaster*, Vasa plays a key role in the localization and translational regulation of germ line specific mRNAs such as *nanos*, *gurken*, and the Oskar protein [61]–[64]. Recent interest in Vasa including in non-model species also indicates that Vasa plays roles in mitosis as well as germ line maintenance and meiosis. The expanding catalogue of Vasa roles and locations beyond the germ line includes multipotent stem cells, embryonic cells, tumors, primordial germ cell specification, stem cell maintenance, cell cycle progression, and piRNA biogenesis (see [65]).

Database searches indicated that *S. mansoni* has at least 33 DEAD-box helicases, consistent with repertoires in other species - the human genome codes for 36 DEAD-box helicases [66], *Saccharomyces cerevisiae* has 26 [50], and 22 are known from *Plasmodium falciparum* [53]. Among these, three *vasa*-like genes were identified and designated *Smvlg1* to *3*. A complement of three *vasa*-like genes for *S. mansoni* conforms with other species where from one to four genes have been identified: humans [67], *D. melanogaster* [19], and *Xenopus* [15] all possess one, the anemone *Nematostella vectensis* possesses two [68], *N. girellae* has three [28], and *C. elegans* has four [17].

Phylogenetic analysis confirmed that *Smvlg1* was a member of the PL10 subfamily of the DEAD-box RNA helicases, although it formed a separate cluster with orthologues from other flatworms that have been classified as *vasa*-like genes. *Smvlg1* was most closely related to ATP-dependent RNA helicases of *S. japonicum* (DDX3X) and *C. sinensis*, and *Smvlg3* appeared closely related to Vasa of *S. japonicum* and another ATP-dependent RNA helicase of *C. sinensis* (DDX3X). *Smvlg2* showed high identity to the DDX3/DED1 helicase of *C. sinensis*, to *N. girellae vasa-like gene 2* (*Ngvlg2*), for which earlier expression and RNAi analyses had confirmed as a *vasa*-like gene [28]. *Smvlg1* and *3* share substantial identity with PL10 (PL10 is termed DDX3 in humans); DDX3 is a DEAD box RNA helicase that promotes mitotic chromosome segregation in somatic human cells [69]. Differences in function have been described between PL10 and Vasa, although there is uncertainty about phylogenetic relationships among flatworm *vasa*-like genes and their homologues. PL10 is not restricted to the germ line and is expressed in numerous other tissues [70], [71]. Notably, PL10-related DED1 of yeast may be required for translational initiation of all mRNAs [72]. The phylogenetic relationship between Vasa and PL10 suggests that Vasa is derived from an ancestral PL10 related gene that subsequently acquired specificity for the germ line [27], although phylogenetic analysis has not been able to reliably assign flatworm DEAD-box helicases into PL10 or Vasa subfamilies (see [28], [30]). Nonetheless, mapping the exon-intron boundaries corroborated the evolutionary relatedness of *Smvlg1* and *Smvlg3* compared to *Smvlg2*.

Sequence analysis of *Smvlg1* and *Smvlg3* indicated that they shared the 10 conserved motifs characteristics of DEAD-box proteins plus the EARKF motif that is diagnostic of PL10 and Vasa-like sub-groups of the DEAD box helicases [51], [52]. In *Smvlg2*, the Q and EARKF motifs were absent in like fashion to *Ngvlg2* of the *N. girellae* [28], [53]. Furthermore, *Smvlg1* and *Smvlg3* retained conserved Trp and Asp residues proximal to the stop codon. *Smvlg2* has evolved two Asp residues near the stop codon. The presence of Trp, Glu, and Asp residues in close proximity to start and stop codons is common within the Vasa family [56]. Moreover, at the amino-terminus of *Smvlg1* there was a short glycine-rich region that contains five Arg-Gly repeats and one RGG motif. Repetitive RGG motifs and G-rich regions near the amino-terminus are known from Vasa orthologues but not from PL10 enzymes [73]. The RGG repeats are thought to function in RNA binding as well as a site for Arg methylation [74]–[76]. In both *Danio rerio* and *D. melanogaster*, the RGG repeats are required for subcellular localization of Vasa to the nuage structure [21], [77]. Vasa localizes at the subcellular level to polar granules (also variously termed or co-localized with P-granules, P-bodies, nuage, mitochondrial cloud and/or chromatoid bodies), structures that are electron-dense, perinuclear, and rich with ribonucleoproteins [11], [77]–[79]. In *Smvlg3*, asparagine-rich stretches were present in the C-terminal region, similar to other Vasa proteins including the *D. japonica vasa-like gene B* (*DjvlgB*) [30], [74]. PSORT II analysis predicted that *Smvlg1* and *Smvlg3* were nuclear proteins whereas *Smvlg2* was mitochondrial, which is consistent with other species [79].

Developmental expression profiles of *Smvlg1*, *Smvlg2* and *Smvlg3* were similar and all three were expressed in all developmental stages of *S. mansoni* examined. Notably, however, 12 hour old IVLE displayed expression levels strikingly higher than other stages, and expression was elevated in adult females compared to males. When released from the female, the schistosome egg is undeveloped; accordingly, the elevated levels of these *vasa*-like transcripts in IVLE likely reflected higher ratio of germ to soma cells in these undeveloped/underdeveloped eggs compared to the other developmental stages. If so, targeting IVLE with transgenes in order to establish lines of transgenic schistosomes may be a judicious strategy [34]. (Alternatively or in addition, these elevated levels of *vasa*-like transcripts in the developing egg may be of maternal origin [80].) WISH was employed to examine the spatial expression of *Smvlg1*, *Smvlg2*, and *Smvlg3* in the adult schistosomes. Elevated expression was observed in the posterior ovary where mature oocytes develop [81]. Tissue-specific, transcriptomic analysis of *S. mansoni* also indicates up-regulation of *Smvlg1* and *Smvlg2* in ovary and *Smvlg2* in the testis [82] (not shown). Up-regulation of *vasa*-like genes in mature oocytes has been shown in other species [67], [80].

Expression of Vasa in late oogenesis indicates that Vasa has a role as a maternal factor for the determination of germ lineages [80]. In *D. rerio*, *Xenopu*s species, *C. elegans*, and *D. melanogaster*, primordial germ cells are specified by maternally inherited cytoplasmic determinants that involve Vasa-related proteins. By contrast, in the mouse, primordial germ cells are induced during gastrulation [83]–[85]. The Vasa homolog MVH is found in primary oocytes but not in mature oocytes of the mouse [86]. The timing of developmental expression of *vasa* like genes in some Lophotrochozoans is known to take place continuously - during embryogenesis, after embryogenesis and continuously in adults [87]. Accordingly, it is also feasible that the schistosome *vasa*-like genes may not be expressed only in germ cells but also in somatic cells since all three are expressed in numerous developmental stages. As noted, the schistosome *vasa*-like genes may be valuable markers in functional genomics of schistosome to monitor introduction of transgenes into the schistosome germ line, but this pattern of expression may impact on the potential use of *vasa*-like genes as markers for germ cell identification and transformation [34], [88]. This potential impediment notwithstanding, WISH corroborated expression of *Smvlg* genes in germ cell rich tissues and organs of the adult female schistosome.

Deeper investigation of the cell and molecular biology of *Smvlg1*-*3* should enhance our understanding of oogenesis, reproduction and the germ line in schistosomes. Gender dissimilarities in essentiality of Vasa, e.g. in mice null for Vasa, males are sterile whereas female are not, while in the hermaphroditic monogenean fluke *N. girellae* silencing of *vasa*-like genes results in loss of germ cells [12], [28], will be relevant to this investigation. Finally, and following up these reports of these critical roles of *vasa* in diverse species, DEAD-box RNA helicases have been identified as essential for schistosome survival [7], and hence the potential of the *vasa*-like gene products as intervention targets in schistosomes is also worthy of consideration.

## Supporting Information

Figure S1 Multiple sequence alignment of 33 *Schistosoma mansoni* DEAD-box RNA helicases. Shown is an alignment of all the motifs conserved in DEAD-box helicases. Above the alignment is a schematic representation of ten motifs conserved in DEAD box helicases; the EARKF motif, is additionally conserved in Vasa and PL10 DEAD-box helicases. Capital case letters indicate amino acids known to be highly conserved (in >80% enzymes examined) whereas lower case letters represent amino acids conserved in 50–79% of helicases. The capital case letters of the Q motif indicate amino acids conserved between 49–99% of helicases. The lower case letters represent groups of amino acids, where a represents an aromatic residue, c is a charged residue, o is an alcohol, and l is an aliphatic residue. Accession numbers for the aligned DEAD-box RNA helicases were taken from GeneDB.
(TIF)

Table S1 Sequences of 33 putative DEAD-box helicases deduced from the genome sequence of *Schistosoma mansoni*. Genome database (http://www.genedb.org/Homepage/Smansoni) accessions for *S. mansoni* orthologues are shown on the left. Closest matches for schistosome enzymes with other species are shown on the right along with protein name, species, GenBank database accession number, *e*-value, and percent identity (I). Highlighted in red are the *S. mansoni* DEAD-box proteins homologous to PL10/Vasa-like proteins.
(TIF)
