# Supplementary figures and images for: Vasa-Like DEAD-Box RNA Helicases of Schistosoma mansoni
Source: PLoS Negl Trop Dis. 2012 Jun 12;6(6):e1686. doi: 10.1371/journal.pntd.0001686 (PMC3373655; doi:10.1371/journal.pntd.0001686)

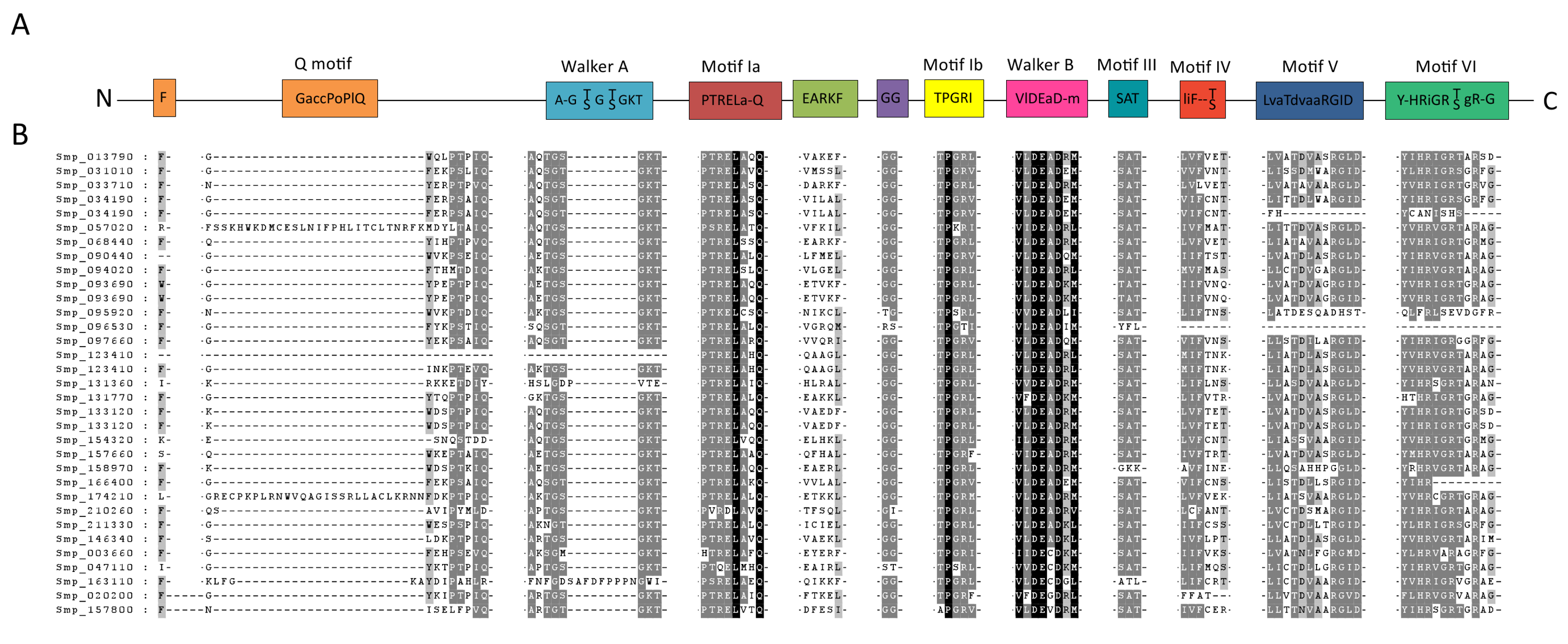

Supplement: Figure S1 — Multiple sequence alignment of 33 Schistosoma mansoni DEAD-box RNA helicases. Shown is an alignment of all the motifs conserved in DEAD-box helicases. Above the alignment is a schematic representation of ten motifs conserved in DEAD box helicases; the EARKF motif, is additionally conserved in Vasa and PL10 DEAD-box helicases. Capital case letters indicate amino acids known to be highly conserved (in >80% enzymes examined) whereas lower case letters represent amino acids conserved in 50–79% of helicases. The capital case letters of the Q motif indicate amino acids conserved between 49–99% of helicases. The lower case letters represent groups of amino acids, where a represents an aromatic residue, c is a charged residue, o is an alcohol, and l is an aliphatic residue. Accession numbers for the aligned DEAD-box RNA helicases were taken from GeneDB. (TIF) [file pntd.0001686.s001.tif]

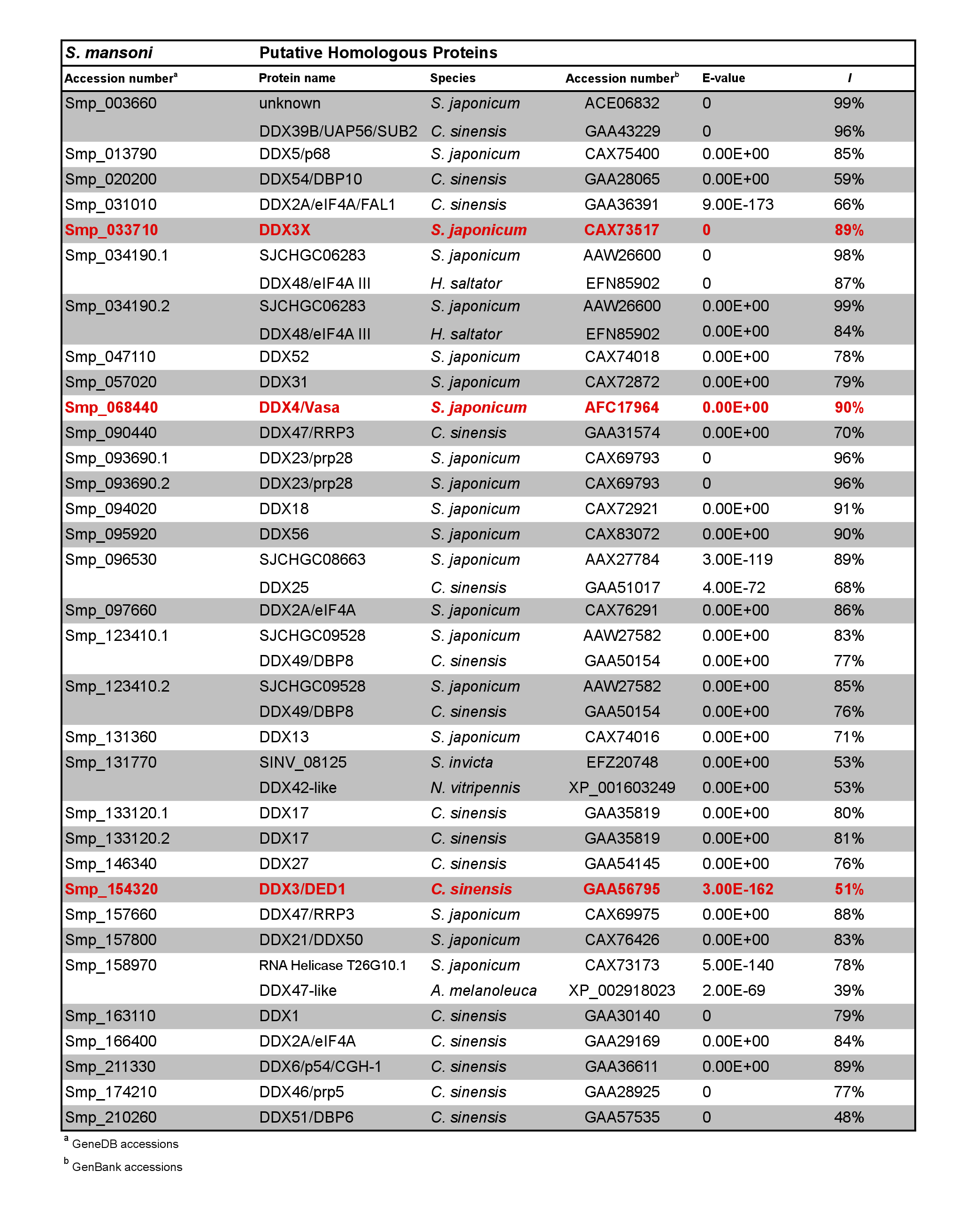

Supplement: Table S1 — Sequences of 33 putative DEAD-box helicases deduced from the genome sequence of Schistosoma mansoni. Genome database (http://www.genedb.org/Homepage/Smansoni) accessions for S. mansoni orthologues are shown on the left. Closest matches for schistosome enzymes with other species are shown on the right along with protein name, species, GenBank database accession number, e-value, and percent identity (I). Highlighted in red are the S. mansoni DEAD-box proteins homologous to PL10/Vasa-like proteins. (TIF) [file pntd.0001686.s002.tif]
